# LiNbO_3_ dynamic memristors for reservoir computing

**DOI:** 10.3389/fnins.2023.1177118

**Published:** 2023-04-11

**Authors:** Yuanxi Zhao, Wenrui Duan, Chen Wang, Shanpeng Xiao, Yuan Li, Yizheng Li, Junwei An, Huanglong Li

**Affiliations:** ^1^School of Instrument Science and Opto Electronics Engineering, Beijing Information Science and Technology University, Beijing, China; ^2^Department of Precision Instrument, Center for Brain Inspired Computing Research, Tsinghua University, Beijing, China; ^3^China Mobile Research Institute, Beijing, China; ^4^School of Chemistry and Chemical Engineering, Jining Normal University, Ulanqab, China; ^5^Inner Mongolia Qingmeng Graphene Technology Co., Ltd, Ulanqab, China; ^6^Chinese Institute for Brain Research, Beijing, China

**Keywords:** LiNbO3, memristors, volatile memristors, dynamic memristors, reservoir computing

## Abstract

Information in conventional digital computing platforms is encoded in the steady states of transistors and processed in a quasi-static way. Memristors are a class of emerging devices that naturally embody dynamics through their internal electrophyiscal processes, enabling nonconventional computing paradigms with enhanced capability and energy efficiency, such as reservoir computing. Here, we report on a dynamic memristor based on LiNbO_3_. The device has nonlinear I-V characteristics and exhibits short-term memory, suitable for application in reservoir computing. By time multiplexing, a single device can serve as a reservoir with rich dynamics which used to require a large number of interconnected nodes. The collective states of five memristors after the application of trains of pulses to the respective memristors are unique for each combination of pulse patterns, which is suitable for sequence data classification, as demonstrated in a 5 × 4 digit image recognition task. This work broadens the spectrum of memristive materials for neuromorphic computing.

## Introduction

The basic workings of the digital computer remain much the same as they were decades ago: information is represented as binary bits as embodied in the steady states of transistors and processed in a quasi-static way. As transistor scaling that has driven advances in digital computing at an exponential rate since 1960s is approaching its physical limits, it is now delivering performance improvements at a slower pace. A promising alternative is performing computing based on intrinsic device dynamics, such that each device functionally replaces elaborate digital circuits (Zhang et al., 2019; Kumar et al., 2022).

Memristors are a class of emerging devices that naturally embody dynamics through their internal electrophysical processes (Chua, 1971; Strukov et al., 2008; Zhang et al., 2019; Kumar et al., 2022). As their name suggests, memristors are resistors with memory, exhibiting history-dependent resistances. Memristors can be classified into two types according to the retention of their memory: volatile memristors, whose memory disappears at zero bias, and nonvolatile memristors, whose memory is retained at zero bias. Of these two types of memristors, volatile memristors, with their electrically excited resistance states being dynamically evolvable over short periods of time after the electrical stimulations have ceased (short-term memory), are of particular interest for emulating biological neurons in the domain of neuromorphic computing that draws inspiration from the biological neural networks to address the limitations of the conventional digital computing.

Reservoir computing (RC) is an artificial recurrent neural network-based computing framework that maps input time-series data to higher dimensional spaces through the dynamics of a collection of randomly interconnected nonlinear nodes called a reservoir (Jaeger, 2001; Maass et al., 2002). The interconnection weights in the reservoir remain fixed. The states of the reservoir are read out through an output layer for classification. Only the connections in the output layer are required to be trained. Because of this training procedure, RC is easy to use and capable to generalize.

Traditionally, the reservoir uses a large number of nodes to create complex recurrent dynamics. Appeltant et al. (2011) proposed a novel method that reduces the usually required large number of elements to a single nonlinear node with delayed feedback. Since then, there has been extensive research interest in implementing RC in electronic and optical hardware, of which memristive implementations are very attractive because even a single volatile memristor can be used as a reservoir (Du et al., 2017; Moon et al., 2019; Zhong et al., 2021; Liu et al., 2022a,b; Zhong et al., 2022). The key rationale is that the collection of interconnected nodes that form delayed feedback loop in the spatial domain is equivalent to a number of dynamically coupled virtual nodes in the temporal domain, generated by time-multiplexing a single physical dynamic node (e.g., volatile memristor).

LiNbO_3_ (LNO) is a widely used material in integrated and guided-wave optics (Zhu et al., 2021) and an emerging material for neuromorphic computing (Wang et al., 2017; Yakopcic et al., 2017; You et al., 2019; Huang et al., 2021; Liang et al., 2021; Tong et al., 2021). Tong et al. (2021) used the nonvolatile ferroelectric polarization in crystalline LNO gate dielectric for tuning the channel conductance that represents synaptic weight. Two-terminal LNO devices have also been shown to exhibit nonvolatile resistive switching behavior that mimics long-term synaptic plasticity (Wang et al., 2017; Yakopcic et al., 2017; You et al., 2019; Huang et al., 2021; Liang et al., 2021). In this work, we demonstrate that is also a useful material for making volatile memristors. Comprehensive electrical measurements are conducted to reveal the filamentary nature of the resistive switching and the short-term memory behavior in the device. Taking advantage of the short-term memory effect, we demonstrate a memristive reservoir by time multiplexing the device for digit recognition.

## Results and discussion

**Figure 1:**
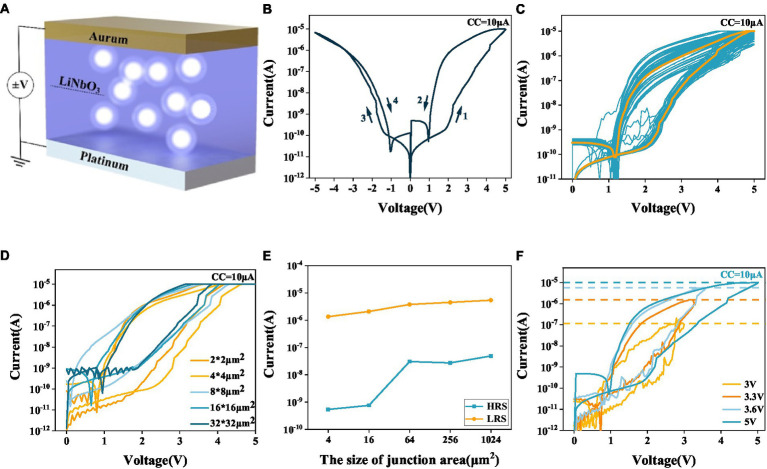
**(A)** Schematic structure of the Pt/LNO/Au memristor. **(B)** Typical I-V characteristics of the LNO memristor. **(C)** Fifty-cycle endurance test of the LNO memristor. **(D)** I-V characteristics of five LNO memristors with different sizes. **(E)** The currents passing through five LNO memristors with different sizes in their respective LRSs and HRSs. **(F)** I-V characteristics obtained by varying the stop voltage during the sweeping.

Figure 1A shows the schematic structure of the Pt/LNO/Au memristor (see Methods). Figure 1B shows a typical I-V curve of the device obtained under cyclic quasi-DC voltage sweeping with the compliance current (CC) set to 10 μA (see Methods). The device can be bi-directionally switched from a high resistance state (HRS) to a low resistance state (LRS) when the voltage of either polarity exceeds each respective threshold value. When the voltage intensity decreases to some hold value (smaller than the threshold value), the device undergoes spontaneous transition from the LRS to HRS, indicating that the device is a volatile memristor. The I-V characteristics of the device are nonlinear, exhibiting nonlinearity (defined as the ratio between the conductance read at the stop voltage 5 V and that read at a half) of about 10^2^ in the positive direction and 2 in the negative direction. Figure 1C shows the I-V curves obtained under consecutive positive voltage sweepings. It is seen that the volatile switching behavior under the CC of 10 μA is reproducible in 50 cycles.

To gain further insights into the switching mechanism, LNO memristors with different sizes are fabricated. Figure 1D shows the I-V curves for five devices obtained under positive voltage sweepings. Though the sizes are different, all devices exhibit volatile switching characteristics. Regardless of the size of the device, the device switches to its LRS at around 2 V. Figure 1E shows the dependence of the LRS and HRS currents (read at 5 V) on device size. While the HRS current increases with increasing device size, the LRS current remains relatively unchanged. This is an indication of the filamentary nature of the volatile switching. Figure 1F shows the I-V curves of the device (2 × 2 μm^2^) obtained under positive voltage sweepings with different stop voltages. It can be seen that the LRS current (read at 2.2 V) increases with the stop voltage, which can be understood as due to the formation of thicker conducting filament under stronger voltage.

**Figure 2:**
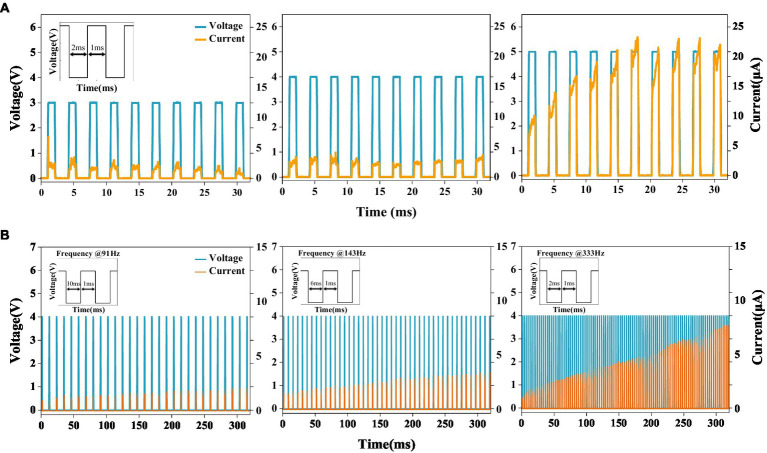
**(A)** Evolutions of the currents passing through the memristor upon receiving trains of pulses with different pulse amplitudes. **(B)** Evolutions of the currents passing through the memristor upon receiving trains of pulses with different pulse frequencies.

Pulse train measurements are also performed. As shown in Figure 2A, under the pulse frequency of 333 Hz and intensity of 3 V, the current response to each voltage pulse remains almost unchanged. This is because the filament grown under the voltage pulse has been ruptured spontaneously during the pulse interval; or in other words, the memory of the device has faded away over the interval. When the pulse intensity increases to 4 V while keeping its frequency unchanged, the current response becomes stronger as the number of pulses increases. By further increasing the intensity to 5 V, the current response is dramatically enhanced because of the highly nonlinear I-V relationship but saturates after the first few pulses. We also investigate effect of pulse frequency on the switching behavior. As shown in Figure 2B, under the pulse frequency of 91 Hz and intensity of 4 V, the current increases almost linearly with each pulse. This linearity can be observed more clearly under higher pulse frequencies. The increasing current response with each pulse under the condition of large pulse intensity or high pulse frequency is an indication that the consecutive switching events are dynamically coupled. This can be understood as due to the short-term memory effect of the volatile memristor. To be more specific, though filament dynamically decays during the pulse interval, large pulse intensity and high pulse frequency make it more likely for a fraction of the filament to be still present at the arrival of the immediately following pulse. Therefore, filament re-growth does not start from scratch but from the last residual, resulting in increasing current response.

**Figure 3:**
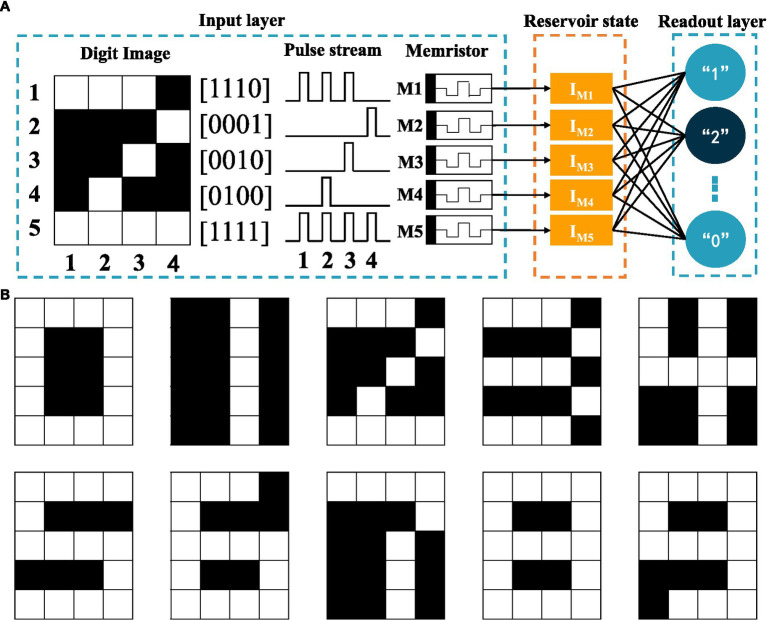
**(A)** Schematic of the memristive RC system for digit recognition. **(B)** Binary images for digit 0–9.

**Figure 4:**
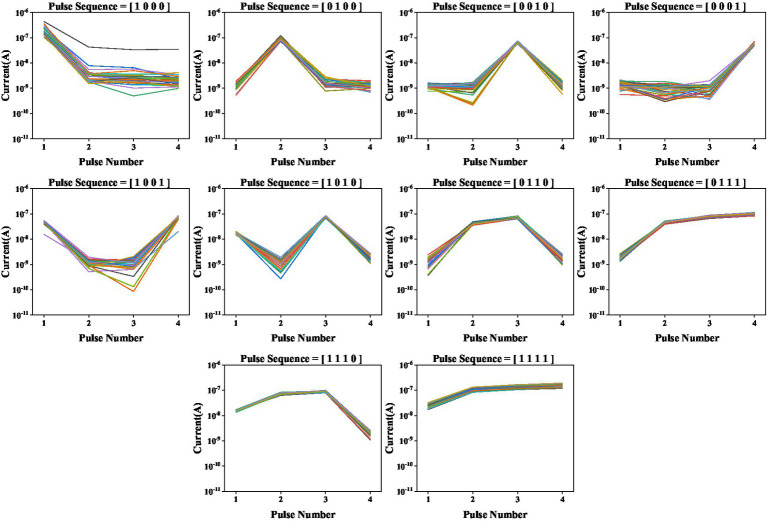
Evolutions of the currents passing through the memristor upon receiving trains of pulses with different patterns. Measurements are conducted for 30 times.

**Figure 5:**
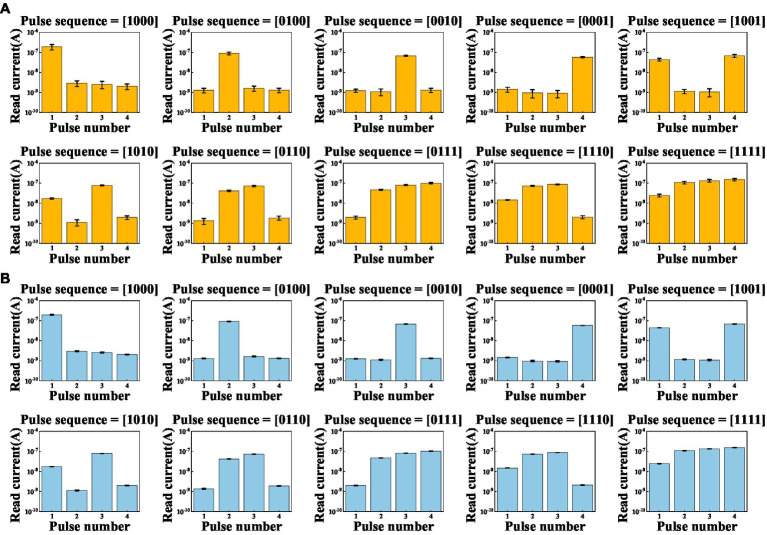
Evolutions of the mean currents passing through a memristor upon receiving trains of pulses with different possible patterns, where **(A)** error bars are standard deviations, **(B)** error bars are standard errors of the means, over 30 trials.

Based on the dynamic nature of the volatile LNO memristor, we create a RC system and demonstrate the digit recognition task. As shown in Figure 3A, the white and black binary pixel information of a 5 × 4 grey-scale image are encoded in voltage pulses of 5 V and 0 V, respectively. To ensure dynamical coupling between consecutive multiplexing events, the pulse width is chosen to be 1 ms and the pulse interval is chosen to be 2 ms that is short enough to prevent the memory of the device from completely fading away over the period. Each row as a four-timeframe input stream is fed into a memristor. In this way, a spatial image is transformed to time series data. As shown in Figure 3B, there are in total 10 different row patterns in these 5 × 4 binary digit images. We explicitly show in Figure 4 the temporal evolutions of the states of a LNO memristor upon receiving pulse trains encoding these row patterns. Figure 5 shows the evolution of the mean currents passing through this memristor upon receiving trains of pulses with different possible patterns. Standard deviations and standard errors of the means are obtained over 30 trials.

**Figure 6:**
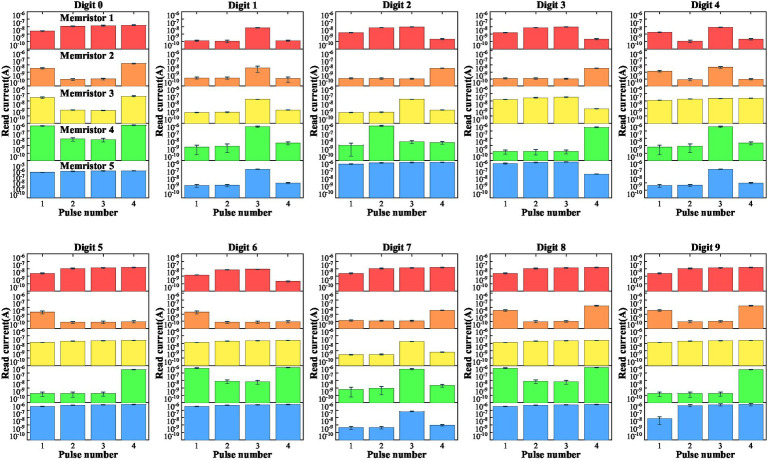
Evolutions of the mean currents passing through five memristors upon receiving trains of pulses with different possible patterns. The error bars are standard deviations over 30 test trials.

For digit recognition, only five LNO memristors are used, each serving as a component reservoir for mapping the corresponding input row data into high-dimensional space. By collectively processing the temporal features in these five input pulse streams, information of the image is extracted. After receiving the input streams, the collective reservoir state (i.e., the current values obtained from five memristors at the last time point) is dependent on the input temporal patterns and therefore can be used to analyze the input. Figure 6 shows the mean current evolutions of these five memristors in recognizing these 10 digits. Table 1 lists the *p*-values for Hotelling’s *T*-square tests that show differences between all collective reservoir states corresponding to different digit images.

**Table 1:** *p*-values for Hotelling’s T-square tests that show differences between all collective reservoir states corresponding to different digit images.

*p*-value	Digit0	Digit1	Digit2	Digit3	Digit4	Digit5	Digit6	Digit7	Digit8	Digit9
Digit0		4.206e-45	4.659e-66	5.396e-47	4.062e-43	1.461e-50	9.325e-57	2.356e-42	3.935e-27	3.388e-30
Digit1	4.206e-45		2.506e-39	2.180e-61	1.391e-63	5.248e-50	4.807e-47	1.032e-59	3.450e-51	5.387e-45
Digit2	4.659e-66	2.506e-39		2.438e-40	1.865e-44	5.766e-82	1.526e-76	1.307e-35	8.767e-79	3.801e-51
Digit3	5.396e-47	2.180e-61	2.438e-40		4.080e-67	1.817e-55	1.005e-50	1.777e-55	2.722e-54	7.244e-45
Digit4	4.062e-43	1.391e-63	1.865e-44	4.080e-67		1.287e-38	1.633e-38	4.218e-71	7.733e-41	1.902e-34
Digit5	1.461e-50	5.248e-50	5.766e-82	1.817e-55	1.287e-38		1.689e-47	3.968e-47	3.670e-45	2.439e-26
Digit6	9.325e-57	4.807e-47	1.526e-76	1.005e-50	1.633e-38	1.689e-47		6.242e-49	2.617e-45	2.835e-40
Digit7	2.356e-42	1.032e-59	1.307e-35	1.777e-55	4.218e-71	3.968e-47	6.242e-49		3.205e-47	2.145e-42
Digit8	3.935e-27	3.450e-51	8.767e-79	2.722e-54	7.733e-41	3.670e-45	2.617e-45	3.205e-47		1.712e-24
Digit9	3.388e-30	5.387e-45	3.801e-51	7.244e-45	1.902e-34	2.439e-26	2.835e-40	2.145e-42	1.712e-24	

For these pairs of digits, we see that their collective reservoir states are significantly different, verifying the reservoir’s ability to separate them. The output layer applies a transformation to the reservoir state through a weight matrix and subsequent nonlinear activations. The training is implemented in software. Specifically, the softmax function is used as the activation function to normalize the output of the network to a probability distribution of 10 possible outcomes. The current values obtained from five memristors at the last time point are fed into the readout network for digit recognition. The weight matrix of the size of 5 × 10 is trained in a supervised way using the Moore-Penrose pseudo-inverse method. The cost is calculated from the categorical cross-entropy.

## Conclusion

In summary, we have demonstrated a Pt/LiNbO_3_(LNO)/Au volatile memristor whose resistive switching has a filamentary nature according to size-dependent resistance measurements. The device exhibits short-term memory as revealed from the pulse train measurements. The pulse-induced switching events can be dynamically coupled by using relatively strong pulses and short pulse intervals. By time multiplexing, a single LNO memristor serve as a reservoir whose pattern-sensitive dynamic responses to pulse trains can be used for sequence data classification. We have used the dynamic characteristics of the volatile memristor in reservoir computing to achieve digit recognition. This work provides a new vision of LiNbO_3_ for neuromorphic electronic devices beyond optics.

## Experimental section

### Device fabrication

Pt (30 nm)/LiNbO_3_(100 nm)/Au (30 nm) were fabricated on a SiO_2_/Si substrate by photolithography (Karl Suss MA8/BA8 Mask Aligner) and magnetron sputtering (AJA sputtering system), followed by a lift-off process. The pressure during sputtering of all three materials is 3 mTorr, the power for depositing two electrode films is 50 W, and the power for depositing LiNbO_3_ films is 70 W. All of these deposition processes are operated under room temperature in an ultrapure argon atmosphere.

### Electrical measurements

The device measurements were carried out using a semiconductor parameter analyzer (B1500A, Agilent), and a waveform generator/fast measurement unit (B1530A, Agilent). For I-V and pulsed tests, Au is connected to the tip and Pt is grounded.

## Data availability statement

The raw data supporting the conclusions of this article will be made available by the authors, without undue reservation.

## Author contributions

YZ and WD contributed equally to this work. HL and WD conceived the idea and supervised the project. YZ and WD performed the device fabrication and measurements. YZ, CW, and WD performed the statistical analyses. YZ, WD, and HL wrote this manuscript with input from all authors.

## Conflict of interest

JA was employed by Inner Mongolia Qingmeng graphene Technology Co., Ltd.

The remaining authors declare that the research was conducted in the absence of any commercial or financial relationships that could be construed as a potential conflict of interest.

## Publisher’s note

All claims expressed in this article are solely those of the authors and do not necessarily represent those of their affiliated organizations, or those of the publisher, the editors and the reviewers. Any product that may be evaluated in this article, or claim that may be made by its manufacturer, is not guaranteed or endorsed by the publisher.

## Funding

The authors acknowledge funding from STI 2030—Major Projects (2021ZD0200300), National Natural Science Foundation (grant nos. 61974082), Youth Elite Scientist Sponsorship (YESS) Program of China Association for Science and Technology (CAST) (no. 2019QNRC001) and Ministry of Education Key Laboratory of Luminescence Analysis and Molecular Sensing (Southwest University).
